# Herbivore Impacts on Marsh Production Depend upon a Compensatory Continuum Mediated by Salinity Stress

**DOI:** 10.1371/journal.pone.0110419

**Published:** 2014-10-13

**Authors:** Jeremy D. Long, Laura D. Porturas

**Affiliations:** Biology Department and Coastal & Marine Institute Laboratory, San Diego State University, San Diego, California, United States of America; Institute of Ecology and Biodiversity, Chile

## Abstract

Plant communities are disturbed by several stressors and they are expected to be further impacted by increasing anthropogenic stress. The consequences of these stressors will depend, in part, upon the ability of plants to compensate for herbivory. Previous studies found that herbivore impacts on plants can vary from negative to positive because of environmental control of plant compensatory responses, a.k.a. the Compensatory Continuum Hypothesis. While these influential studies enhanced our appreciation of the dynamic nature of plant-herbivore interactions, they largely focused on the impact of resource limitation. This bias limits our ability to predict how other environmental factors will shape the impact of herbivory. We examined the role of salinity stress on herbivory of salt marsh cordgrass, *Spartina foliosa*, by an herbivore previously hypothesized to influence the success of restoration projects (the scale insect, *Haliaspis spartinae*). Using a combination of field and mesocosm manipulations of scales and salinity, we measured how these factors affected *Spartina* growth and timing of senescence. In mesocosm studies, *Spartina* overcompensated for herbivory by growing taller shoots at low salinities but the impact of scales on plants switched from positive to neutral with increasing salinity stress. In field studies of intermediate salinities, scales reduced *Spartina* growth and increased the rate of senescence. Experimental salinity additions at this field site returned the impact of scales to neutral. Because salinity decreased scale densities, the switch in impact of scales on *Spartina* with increasing salinity was not simply a linear function of scale abundance. Thus, the impact of scales on primary production depended strongly upon environmental context because intermediate salinity stress prevented plant compensatory responses to herbivory. Understanding this context-dependency will be required if we are going to successfully predict the success of restoration efforts and the ecological consequences of anthropogenic disturbances.

## Introduction

Environmental stress can disturb communities directly by reducing survivorship or forcing migrations, or indirectly by altering the traits responsible for determining how species interact [1]–[5]. An important plant trait that can be affected by stress is the ability to compensate for herbivory [6]. In a foundational study, Maschinski and Whitham (1989) proposed the Compensatory Continuum Hypothesis whereby plant compensatory responses can vary in sign and strength, largely via environmental factors. The discovery that compensatory responses to herbivory can be shaped by the environment profoundly influenced our understanding of plant-herbivore interactions [7], [8]. However, most studies focused on the effect of resource limitation on compensatory responses [8]. Indeed, a well-cited revision of the Compensatory Continuum Hypothesis, the Limiting Resource Model, focused on the primacy of the level and identity of the limiting resource [9]. The roles of other environmental stressors on herbivory remain unclear. If compensatory responses generally fall along a continuum determined by environmental stress, then understanding this complexity will become important to successfully predicting the consequences of anthropogenic stressors like pollution and climate change.

Although there is emerging evidence that compensatory responses vary with multiple environmental contexts, we have an incomplete appreciation for the role of environmental stress other than resource limitation. For example, overcompensation appears more commonly for monocot herbs when nutrients are not limiting but overcompensation is more common for dicots under nutrient limitation [8]. Water-stress can also determine compensatory responses and the consequences of herbivory ([10]–[13] but see [14]–[16] for exceptions). In most of these cases, water stress leads to reduced compensatory responses. Unfortunately, the consequences of other common stressors, like salinity, are poorly known. Although the immediate consequences of salinity stress may largely result from changes in water uptake, salinity stress can create unique problems for plants that influence their ability to compensate for herbivory [6], [17]. For example, water and salinity stress may have very different impacts on natural communities because the former increases soluble nitrogen and nutritional quality of plants [14], [15], [18], whereas the latter can decrease nutritional quality [19], [20]. Also, salinity stress may influence compensatory responses because of negative, salt-specific effects on hormonal regulation, photosynthesis, and cellular function [17]. Furthermore, salinity stress might directly suppress herbivore performance via increases in plant tissue salt concentrations [21]. Thus, previous studies may fail to predict the impact of salinity stress on plant compensatory responses and their consequences because of stress-specific effects on plant physiology.

Because of their distinct gradients in environmental stress and plant distributions [22], [23] and their susceptibility to anthropogenic stressors like climate change, salt marshes represent important habitats where salinity stress may interact with herbivory to influence primary production. Three observations support this hypothesis. First, experimental salinity stress increased the abundance of marsh planthoppers ([24], [25] but see [20] for an exception). Second, palatability of marsh plants depended upon whether plants were collected from high salinity versus low salinity sites [26]. Third, salt addition stress magnified the negative impact of snail herbivory on *Spartina* biomass [27], perhaps because salt impaired the ability of *Spartina* to tolerate herbivory or increase resistance after attack [28]. Taken together, these studies suggest that salinity stress may shape the impact of herbivores on marsh plants and that the synergy of these factors may contribute to marsh dieback [27]. Given that climate change is expected to alter soil salinities via changes in sea level and precipitation [29], [30], there is a pressing need to understand the impact of salinity stress on marsh herbivory.

We examined the impact of salinity stress on top-down control of Pacific cordgrass, *Spartina foliosa*, by the specialist scale insect, *Haliaspis spartinae*. Scale insects are common pests that can reduce the growth and survival of plants [31]–[33]. Although no study has experimentally tested the impact of *H. spartinae* on cordgrass, it has been implicated in the failure of some restoration projects to achieve desired outcomes [34], [35]. These authors noted that, “In 1992, where the highest densities of *Haliaspis* occurred, cordgrass was shorter than in previous years and senesced unusually early in the season” [34]. Because cordgrass height is one measure of restoration success (in part because endangered birds require tall plants to make nests [35]), it was hypothesized that the scales may prevent successful restoration of these sensitive habitats [34], [35]. Thus, an improved understanding of scale impacts and how these change with environmental conditions could lead to improved restoration outcomes.

We tested for a causative relationship between scales and plant performance, and the role of salinity stress on scale herbivory, by manipulating scale presence and salinity in a series of mesocosm and field studies. We predicted that 1) scales would exert the strongest negative impacts on *Spartina* at intermediate salinities where compensatory responses would be impaired, and 2) further stressing *Spartina* with high salinities would weaken scale impacts.

## Methods

We conducted three major experiments to examine the impact of scale insects (*Haliaspis spartinae*, hereafter scales) on *Spartina foliosa* stems (hereafter *Spartina*). First, we used a field experiment to demonstrate that scales can negatively influence *Spartina* performance. Second, we used a mesocosm experiment to examine the effect of reduced salinities on *Spartina*-scale interactions. Finally, we conducted a field experiment to determine the effect of elevated soil salinities on these interactions. The latter two experiments were conducted because 1) the heaviest infestations of scales on *Spartina* (# per stem) occur in the high marsh and 2) high soil salinities in this zone suppress *Spartina* growth [36]. Thus, we hypothesized that top-down control of *Spartina* by scales may be influenced by soil salinity.

Infestations of *Haliaspis*, a *Spartina* specialist, appear highest in Southern California marshes. We have observed heavy infestations of *Haliaspis* from South San Diego Bay to Upper Newport Bay, California. Throughout this range, there is variation within and between marshes in *Haliaspis* densities. Although *Haliaspis* is believed to be indigenous to these marshes, there is some evidence that the distribution and abundance of this scale is expanding. For example, in the late 1980s and early 1990s, *Haliaspis* was abundant in human-constructed marshes but rare or absent in more natural marshes [34], [37]–[39]. Our recent surveys suggest that *Haliaspis* has now expanded into some of these natural marshes at extremely high densities.

The two field experiments were conducted in the high zone of Sweetwater Marsh in South San Diego Bay. Sweetwater is a tidal, hypersaline marsh with soil salinities that frequently exceed 50 ppt. This marsh also has the highest maximum densities of scales per stem that we have encountered during our surveys of southern California marshes (maximum number of scales per stem >3000). Within Sweetwater Marsh, the highest scale densities per stem are observed in the high marsh zone. Salt marsh perennials including *Sarcorcornia virginica*, *Frankenia grandifolia*, *Jaumea carnosa*, *Batis maritima*, and *Suaeda californica* dominate this habitat. This research was permitted by the U.S. Fish and Wildlife Service (SUP 81680-12002) – the organization that manages Sweetwater Marsh.

### Effects of scale removal (2011 Field experiment)

To examine the impact of scales on cordgrass performance, we manipulated scale presence on *Spartina* at Sweetwater Marsh (32.6412°N, 117.1142°W). On 20 May 2011, we randomly selected 20 *Spartina* stems separated by at least 1 m and that contained high densities of mature scales relative to nearby stems (70±19 scales per stem, mean ± SE). Stems were randomly assigned to Scale or No Scale treatments, and scales were removed from half of the stems (No Scale stems, N = 10) by brushing the adaxial surface of leaves with a soft toothbrush. This procedure effectively removes scales because they cluster on the adaxial surface of leaves. As a procedural control, we brushed the abaxial surface of Scale stems. For 20 weeks, we maintained treatments by manually removing scales from No Scale stems with a toothbrush every 1–2 weeks. Because scales have a short larval dispersal period followed by a sessile juvenile and adult period, manual removals effectively create Scale/No Scale treatments [40].

Every 1–2 weeks, we recorded Scale density (# per stem), and *Spartina* senescence, stem height, and seed presence. *Spartina* senescence was determined when the stem’s meristem was completely brown. As a conservative estimate, all plants that had not senesced by our final sampling date (7 October 2011) were assumed to have senesced one week later. Stem height was determined by recording the maximum vertical distance from shoot base to the tip of the longest leaf. Seed presence was recorded as a binomial variable (present/absent). After 20 weeks (7 October 2011), we collected shoots, recorded seed presence, dried the shoots, and recorded final dry shoot biomass. We observed early senescence of many plants in the Scale treatment. These were included in all performance measurements. On 19 August 2011, we observed leaves that had been grazed entirely across their width. Although we did not identify this grazer, this type of damage is consistent with large, vertebrate grazers (e.g. birds). One Scale stem and two No Scale stems contained such damage. To exclude further vertebrate grazing, we placed tomato cages lined with chicken wire (opening ∼3 cm) around all stems.

We compared two measures of growth of Scale and No Scale plants using two-tailed, two-sample t-tests. First, we calculated the proportional change in shoot height during the 20-week experiment. Second, we compared final dry shoot biomass. We used a two-tailed Fisher’s Exact Test to compare the proportion of starting plants that ever produced seeds because 9 of 10 Scale plants did not produce seeds. We compared days to senescence using a two-tailed, two-sample t-test.

### Effects of scale removal and salinity reduction (2011 Mesocosm experiment)

We examined the impact of reduced salinities on *Spartina*-scale interactions using mesocosms because logistical hurdles prevented us from reducing salinities in the field. A limitation of this design is that our mesocosm experiment may have been confounded by other factors. We sought to minimize differences between the field and our mesocosm experiment by conducting mesocosm research outdoors at SDSU’s Coastal & Marine Institute Laboratory that is located 14 km from Sweetwater Marsh. This lab is located adjacent to San Diego Bay (i.e. the same water body from which all organisms were collected). Thus, ambient environmental conditions during our mesocosm studies were likely similar to field conditions.

We collected mud cores (∼15 cm diameter × 15 cm deep) from Sweetwater Marsh that each contained a single *Spartina* stem with scales. We transferred these to the marine lab, and planted each stem-containing mud core into a 2.6 L plastic pot (15 cm height) with holes in the bottom for drainage. Stems were randomly assigned to each of four different treatments created by manipulating Scales (Scale, No Scale) and Salinity (Freshwater, Saltwater). While we recognize that freshwater exposure of southern California *Spartina* is extremely rare, this treatment was included because previous studies showed that salt marsh plants grow optimally in reduced salinity [36]. Most studies of plant stress define stress as any condition that reduces the optimal performance of plants (e.g. [41]). Additionally, ambient soil salinities as low as 15 ppt have been reported for *Spartina foliosa*, especially during high rainfall years [42].

All treatments had 8 replicates, with the exception of the Scale-Saltwater treatment, which had 7. Scales were removed from No Scale stems with a toothbrush and scale treatments were maintained weekly. Stems from each treatment were randomly assigned to shallow pools of a specified salinity treatment (N = 2). Salinity was manipulated by filling the pools to the soil-air interface with either freshwater or saltwater. The saltwater pools were filled with water from the flow-through seawater system that takes in water from San Diego Bay (∼32 ppt). Freshwater pools were filled with water from a tap. Twice per week, we measured the pool salinity with a refractometer (Model RHS-10ATC, Aquatic Eco-Systems Inc.), drained and refilled each pool, and then re-measured pool salinity. We chose to measure the salinity of the pool water rather than the porewater (soil salinity) so that we would minimize disturbance to the soil in the pots. Thus, the magnitude by which our freshwater mesocosm reduced soil salinity is currently unclear. During the water changes, each pot was allowed to drain outside of the pools for ∼30 minutes. All pools were kept outside in full sunlight.

We maintained these treatments for six months (April–October 2011). Every week, we recorded Scale density (# per stem starting 3 May 2011), and *Spartina* senescence and stem height (see above for details). Because the replicates of each treatment were divided amongst two replicate pools, we calculated an average for all replicates within each pool at each time point. During measurements, each pot was allowed to drain outside of the pools for 1–4 hours. We used stem height to calculate the average proportional growth at each time point. These data were analyzed with a Repeated Measures ANOVA. We visually inspected our data and the resulting covariance matrix to confirm that they met the assumptions of this test. However, we were concerned that our data violated the sphericity assumption, perhaps because of the high number of repeated measurements, so we applied a Greenhouse-Geisser correction to this analysis (ε = 0.061). We compared the number of days to senescence using ANOVA. Both ANOVA analyses examined the effect of the fixed factors of Scales (Scale, No Scale) and Salinity (Freshwater, Saltwater) on the response variable.

### Effects of scale removal and salinity addition (2012 Field experiment)

To examine the influence of elevated salinity on the top-down control of *Spartina* by scales, we conducted a manipulative experiment at Sweetwater Marsh during 2012. We selected two sites in March 2012 [hereafter North (32.6412°N, 117.1142°W) and South (32.6388°N, 117.1099°W)]. These sites were selected because of their different elevations (+1.6 m and +2.1 m above Mean Lower Low Water, respectively). Also, we observed negative effects of scales on *Spartina* at the North site during the 2011 experiment. At each site, we randomly selected 1×1 m plots that contained *Spartina* and were separated by at least 1 m. Plots were randomly assigned to a Salinity treatment (Ambient or Elevated) and a Scale treatment (Scale or No Scale). We replicated each of four treatments at both sites (N = 7 or 8; North or South, respectively).

For two months, we pre-treated Elevated Salinity plots with 750 g of solar evaporated sea salt (produced by South Bay Salt Works, Chula Vista, CA) every 2 weeks during low tide. On 26 May 2012, we increased salt additions in Elevated Salinity plots to 1500 g every 2 weeks to create salinities near the maximum levels we have recorded in the field. We collected a single soil core (∼5 cm deep) from each plot for soil salinity analysis on seven dates (31 March, 14 April, 28 April, 12 May, 8 June, 22 June, 6 July, 9 July). Soil salinity was not sampled after 9 July so as to minimize further disturbance to plots. On each of the first seven dates, we sampled plots prior to that day’s salt addition so they reflected salinity differences persisting two weeks after the previous salt addition. In contrast, soil samples from the last date that soil salinities were measured (9 July) were collected 3 d after salt addition. This measurement provided better insight into soil salinity dynamics because the greatest impact of salt additions on soil salinity was observed 3 d after salt additions. We measured soil salinity using a modification of an existing protocol [43]. Briefly, soil cores were weighed, dried for 2 days at 60°C, and then reweighed. Then, we rehydrated cores with the original water volume plus 15 ml deionized water and measured the salinity of the slurry supernatant with a refractometer (Model RHS-10ATC, Aquatic Eco-Systems Inc.). We used these measurements to determine the soil salinity of the original samples.

We marked a single target *Spartina* stem near the center of each Scale and No Scale plot with flagging tape. Scales were removed from No Scale stems using toothbrushes. All stems were initially measured on 26 May 2012, and stems were re-measured and treatments were maintained every 2 weeks thereafter. We measured scale density and recorded the date of senescence. Because previous experiments suggested that plant growth did not extend past September, we stopped this experiment on 14 September 2012.

Salinities were compared using Repeated Measures ANOVA. We excluded the soil salinity data from 6 July 2012 from this analysis because we lost 6 samples during processing and these were not distributed evenly across treatments. We excluded one Enhanced Salinity, Scale plot from the North site because a sample was lost on 6/8/12. We also conducted a three factor ANOVA to examine the effects of Site (North, South), Scales (Scale, No Scale), and Salt (Ambient, Elevated) on the number of days to senescence. We excluded one plant assigned to the North, Scale, Ambient plot because scales never colonized it.

## Results

### Effects of scale removal (2011 Field experiment)

We observed dispersal of juvenile scales beginning in late June and ending in late July 2011 (Fig. S1). During this time, scale densities on Scale plants increased from 154±62 to 771±184 scales per stem (mean ± SE). Although we occasionally observed recruitment onto No Scale plants, the maximum density of scales we observed on No Scale stems was only 124±15 scales per stem. In contrast, we observed an order of magnitude greater maximum scale densities on Scale stems 1531±358 scales per stem. Thus, we effectively maintained differences in scale densities between our treatments.

**Figure 1:**
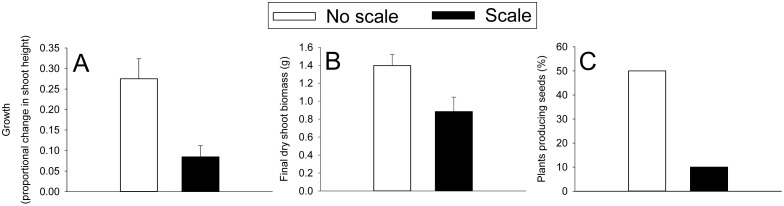
Scale effects on *Spartina* performance during the 2011 field experiment. The three performance measured included A) growth, B) final dry shoot biomass, and C) percentage of plants producing seeds. N = 10. Values are means ± SE.

Scales suppressed *Spartina* shoot height growth by 69% (Fig. 1a; t = 3.381, p = 0.003) and final dry shoot biomass by 37% (Fig. 1b; t = 2.531, p = 0.021). By 7 October 2011, 5 No Scale stems, but only 1 Scale stem, produced seeds. However, the proportion of stems producing seeds was not significantly different at α = 0.05 (Fig. 1c; Fisher’s Exact, p = 0.141).

**Figure 2:**
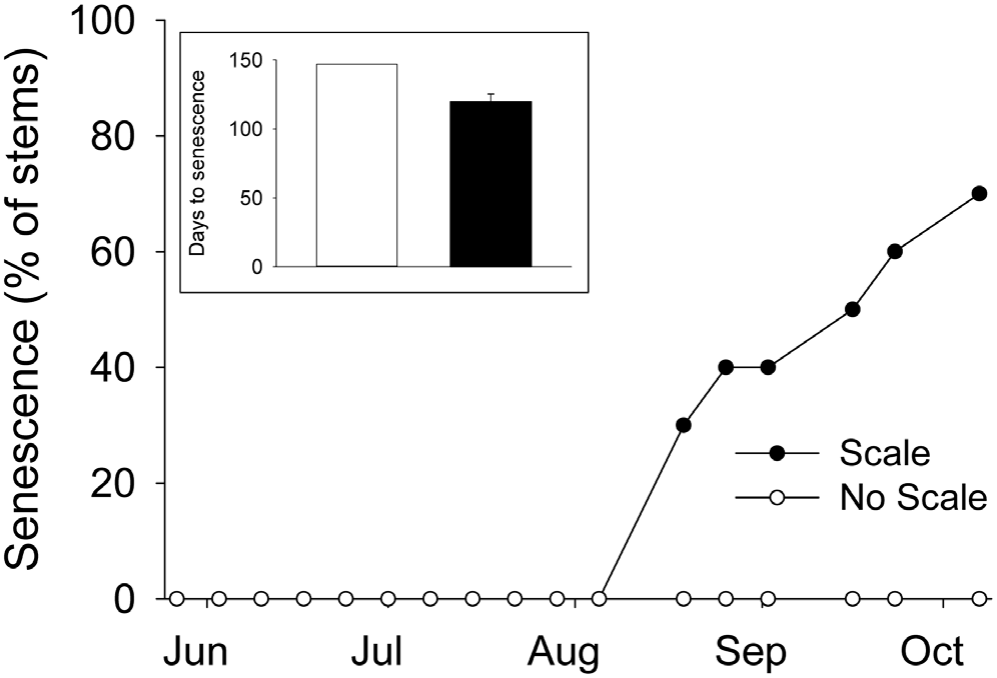
Scale effects on the timing of *Spartina* senescence during the 2011 field experiment. Senescence is reported as the cumulative percentage of stems senesced at each time point. Inset shows the number of days until *Spartina* senescence for No Scale (white bar) and Scale stems (black bar). Stems not senescing at the termination of the experiment were assigned a senescence date of 10/14/11. Because all 10 No Scale stems fell into this category but only 3 Scale stems did, the difference in days to senescence is conservative. This also explains the lack of error bars associated with the No Scale inset. Values in inset are means ± SE.

No plants senesced during the first three months of the 2011 field experiment (Fig. 2). However, 70% of Scale stems senesced between mid-August and the end of the experiment. None of the No Scale stems senesced by the end of the experiment. If we conservatively assume that all No Scale plants senesced a week after the experiment ended, then No Scale stems senesced at least 27 days later than Scale stems (Fig. 2 inset; t = 3.452, p = 0.003). Importantly, the earlier senescence in Scale stems occurred during seed production.

### Effects of scale removal and salinity reduction (2011 Mesocosm experiment)

Salinity of the pools in Freshwater treatments ranged between 0–3.5 ppt, with a mean of 0.3 ppt. In contrast, the salinity in Saltwater treatments was typically an order of magnitude larger (range = 27–59 ppt, mean = 39.8 ppt). *Spartina* in the high marsh can encounter salinities in excess of 80 ppt. Salinity tended to increase in pools after water changes because of evaporation from the pools. Similar to the 2011 field experiment, we saw a major dispersal event of scales in late June/early July (Fig. S2). However, we also saw a pronounced secondary dispersal event in mesocosms that started late in August 2011. Scale densities on Scale stems were higher in Freshwater than Saltwater treatments after the initial dispersal event (Fig. S2). This trend was not evident after the second dispersal event.

Plants senesced earlier in Saltwater treatments (days to senescence = 102±18 and 150±11 for Scale and No Scale stems, respectively) relative to Freshwater treatments (188±0 for both Scale treatments because no Freshwater plant had senesced by the end of the experiment; F_1,4_ = 35.7, p = 0.004). Although there was a trend for scales to cause earlier senescence in Saltwater treatments, neither Scales nor the interaction between Scales and Salinity influenced days to senescence (F_1,4_ = 5.196, p = 0.085).

**Figure 3:**
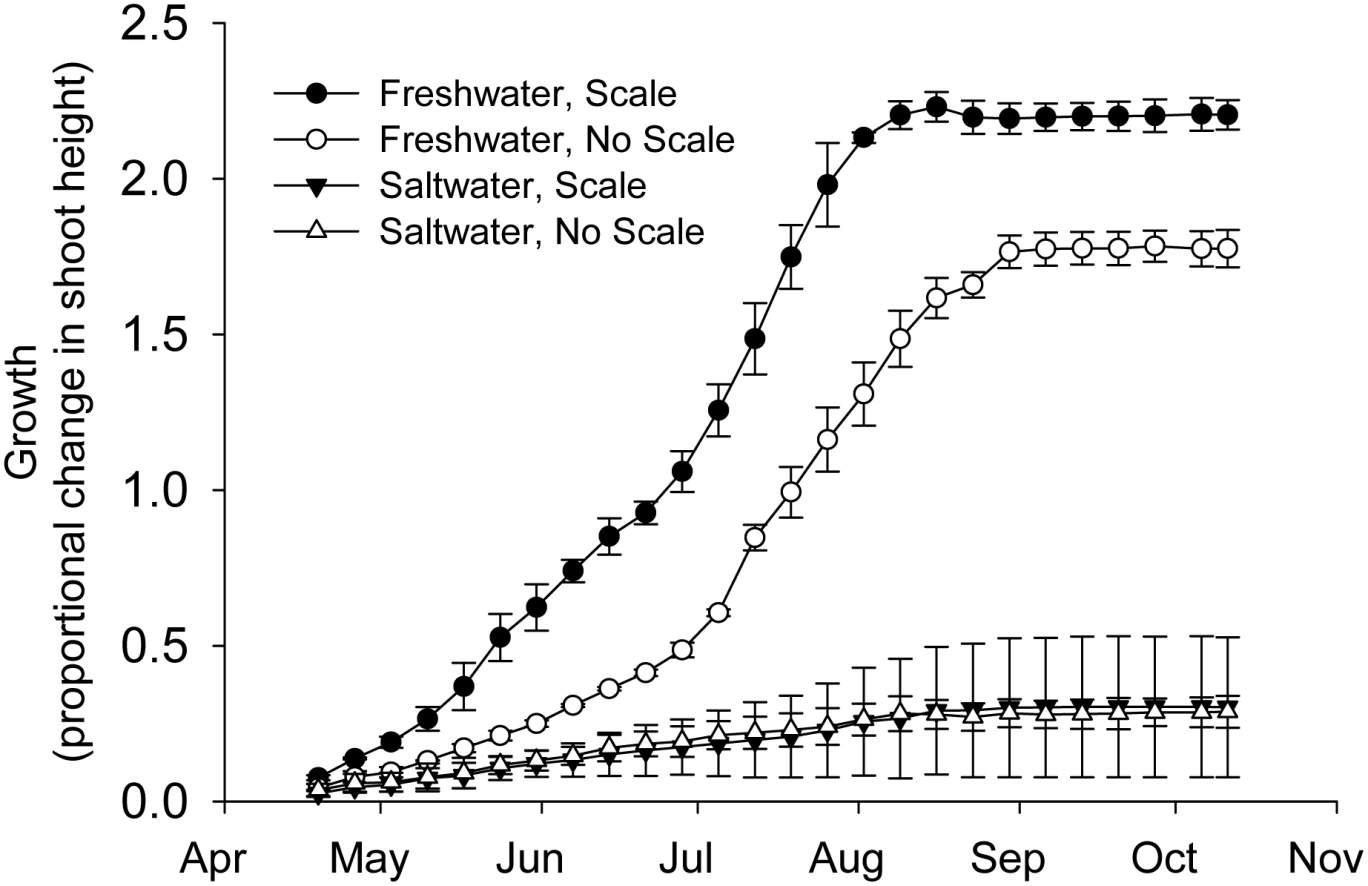
Effects of scales and reduced salinity on *Spartina* growth during the 2011 mesocosm experiment. Growth is reported as the proportional change in shoot height. Overcompensation to scale herbivory in freshwater treatments was absent in saltwater treatments. N = 7–8. Values are means ± SE.

Importantly, Salinity and Scales interacted to influence *Spartina* growth (Fig. 3, Table S1). Scales stimulated *Spartina* growth in Freshwater treatments but had no effect on growth in Saltwater treatments. As expected, *Spartina* growth increased during the experiment.

### Effects of scale removal and salinity addition (2012 Field experiment)

Plots with Elevated Salinity had higher porewater salinities than Ambient plots (F_1,54_ = 21.2, p<0.001; Fig. S3). The increase in soil salinity in Elevated plots was most pronounced three days after adding salt (9 July 2012) relative to two weeks after adding salt (all other dates, Fig. S3). Although overall soil salinities were similar at the North and South sites (F_1,54_ = 0.663, p = 0.419), Elevated Salinity plots contained 22% higher salinity on 9 July 2012 at the North versus the South site (84 vs. 69 ppt, respectively; F_1,28_ = 4.20, p = 0.019). Salinity did not differ with Scale treatment (F_1,54_ = 0.117, p = 0.734). All soil salinity measurements were within the range of soil salinities that can be currently experienced by *Spartina* at Sweetwater (we have measured a maximum soil salinity of 86 ppt).

Compared to the 2011 experiments (Figs. S1–S2), mean scale densities at the beginning of June were lower in 2012 (Fig. S4). For example, although our stems had ∼100 scales per stem at the start of June 2011, mean scale densities in 2012 did not reach 100 scales per stem in any treatment until 6 July 2012. Thus, stems in the 2011 experiments may have experienced greater herbivore pressure than the 2012 experiment. The within-treatment variation of scale abundance increased with time, in part because of early plant senescence within this experiment. Because we removed scales from No Scale plants every two weeks, scale densities were always low on these treatments (Fig. S4).

**Figure 4:**
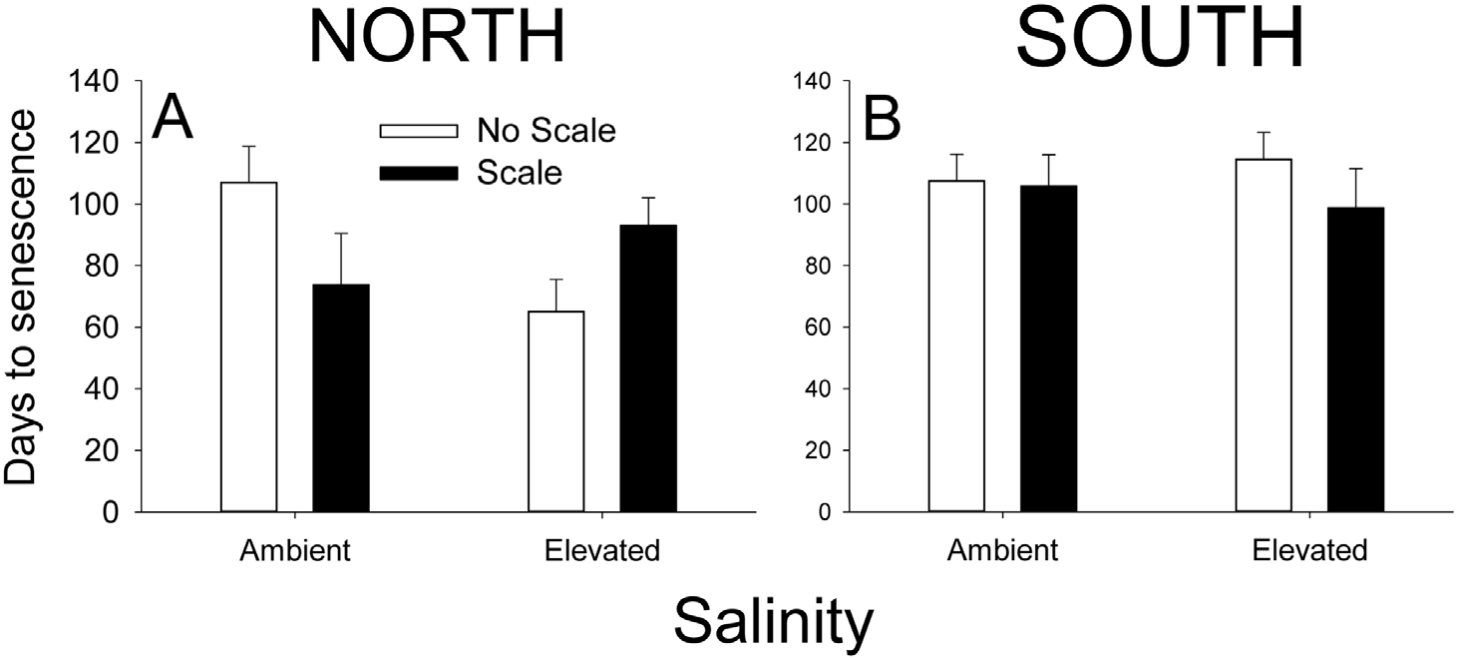
Effects of scales, elevated salinity, and site on *Spartina* senescence during the 2012 field experiment. Senescence is reported as the number of days after the start of the experiment that plants senesced at the North (A) and South Sites (B). N = 7–8. Values are means ± SE.

For days to senescence, we observed a significant three-way interaction between Salt, Scale, and Site (Figure 4, Table S2). To better understand this interaction, we examined the interaction between Salt x Scale at each site separately using the appropriate error term. At the North site, the effect of Scales on senescence depended upon Salinity treatment (F_1,51_ = 7.046, p = 0.011). In contrast, we observed no interaction at the South site (F_1,51_ = 0.437, p = 0.512).

## Discussion

The impact of scales on *Spartina* performance depended strongly upon environmental context. When grown under the lowest salinities (freshwater mesocosms), *Spartina* overcompensated for herbivory by growing taller shoots. However, increasing salinity stress switched the impact of scales from positive to neutral (2011 Mesocosm experiment). Additionally, scales negatively impacted *Spartina* by reducing growth and expediting senescence in the field. These data provide experimental evidence that *Haliaspis* can negatively impact *Spartina* and may have influenced the success of previous restoration efforts [33]. At the hypersaline field site where we observed negative effects of scales in 2011 and 2012, increasing soil salinity further returned the impact of scales to neutral (2012 Field experiment). Interestingly, scale densities and salinity were negatively related suggesting that scale impacts were not simply a linear function of scale abundance.

Previous studies examining the impact of environmental stress on plant compensatory responses largely focused on resource limitation as a stressor [8]. There, low nutrient conditions tended to weaken compensatory responses of monocots. Our study suggests that salinity stress acts similarly to weaken compensatory responses of *Spartina foliosa*. Reduced compensatory abilities with increasing salt stress might be particularly common in dominant salt marsh monocots like *Spartina* and *Juncus*. Salt stress decreases foliar nitrogen in *Spartina* [19], perhaps because of reduced uptake abilities [44], [45]. Taken together, these observations suggest that salinity may alter the impact of marsh herbivores on marsh plants via reductions in compensatory abilities resulting from nutrient limitations. However, this hypothesis should be considered tentative, especially given the numerous exceptions to the general effect of resource limitation on monocots seen previously [8] and because salinity stress can directly impact other aspects of plant physiology [17].

While the interactive effects of environmental stress and herbivores can be complex and difficult to predict [7]–[9], [46], there is some evidence that the impact of marsh herbivory might generally be more intense during intermediate salt stress because of reduced compensatory abilities. A study of a Northwest Atlantic Ocean salt marsh found that snail herbivory on *Spartina alterniflora* was much stronger in plots with experimental salt additions that increased salinity from ∼35 to 57 ppt [27]. We observed positive or neutral effects of scale herbivory at low salinities (<4 to 40 ppt), negative effects at intermediate salinities (∼50–55 ppt), and neutral effects at extremely high salinities (>80 ppt). In contrast, previous research in a Southwest Atlantic Ocean salt marsh found that salt additions attempting to elevate ambient salinity to 60 ppt removed the negative effects of crab herbivores on marsh expansion seen at lower salinities [47]). In that case, crab impacts may have been low in salt addition plots because crabs avoided these areas [47]. Thus, salinity effects on marsh production may also depend upon how salinity directly affects herbivores.

Scales negatively influenced *Spartina* during the 2012 field experiment at only one of two sites (North site), suggesting that scale effects were site-specific. It is unlikely that salinity differences caused this specificity because soil salinity in Ambient Salinity plots showed no clear pattern with site. However, inundation can influence *Spartina* performance and two observations suggest that inundation differed between these sites. First, the North site was ∼0.5 m lower in elevation. Second, soil salinities in Elevated Salinity plots three days after salt additions were much higher at the North site.

In addition to abiotic differences, at least two biotic factors may have influenced the site-specificity of scale effects. First, plant-plant competition may influence plant compensatory responses to herbivores [7], [48]. Both sites have plant communities characteristic of high marsh but the makeup of these communities differed between the sites. *Jaumea carnosa* was more abundant and *Frankenia salina* and *Batis maritima* were less abundant at the site with negative scale effects. High marsh plants can suppress *Spartina* growth [49], and may influence *Spartina*’s ability to tolerate herbivory. Second, scale recruitment happened earlier at the North site, suggesting that scale pressure was greater at this site. Regardless of the mechanism, scales exerted a negative impact on *Spartina* at the site where overall plant senescence occurred earlier further supporting our contention that scale impacts depend upon plant stress.

Because we found the greatest impact of herbivores at intermediate salinities with reduced herbivore densities, our data suggest that the per capita effect of scales on *Spartina* was most negative at intermediate salinities. The realized impact of herbivory under stressful environmental conditions will also be determined by how the stressor impacts higher trophic levels, both directly and indirectly [50]. Unfortunately, the role of predators in this system is poorly known. Two observations suggest that predators may be important. First, potential predators such as beetles and parasitoids are present in these marshes [51], [52]. Second, we observed a distinct secondary dispersal event in our mesocosms that was not present in the field in 2011. The lack of obvious predators in our mesocosms, but not the field, may have allowed for this dispersal event.

Understanding the complex interactions of salt stress and herbivory may help improve restoration strategies. In southern California, where loss of historic salt marshes has been extensive [53], a common restoration approach is to transplant *Spartina* into restoration sites. Perhaps because of the stress associated with transplantation, such approaches have been met with variable success [34], [54], [55]. One possible factor associated with restoration success has been heavy infestations of scale insects [34]. Our results suggest that scales may limit the success of restoration projects at intermediate soil salinities. Understanding this context-dependence may be particularly important given the anticipated changes in salinity with climate change that may alter the top-down control of marshes by herbivores.

## Supporting Information

Figure S1 **Densities of scales during the 2011 field experiment.** Scales were removed from No Scale stems every 1–2 weeks. N = 10. Values are means ± SE.
(TIF)

Figure S2 **Densities of scales during the 2011 mesocosm experiment.** Scales were removed from No Scale stems every week. N = 7–8. Values are means ± SE.
(TIF)

Figure S3 **Soil salinities of plots during the 2012 field experiment.** Because salinity did not depend upon Scale treatment, data were combined for Scale and no Scale stems to facilitate comparisons of the North and South sites. Soil salinity was always measured two weeks after salt additions, except on July 9 when sampling occurred three days after salt additions. Values are means ± SE.
(TIF)

Figure S4 **Densities of scales on stems during the 2012 field experiment.** To highlight site and salt effects, data are shown separately for Scale (A) and No Scale (B) stems. Values are means ± SE.
(TIF)

Table S1 **ANOVA table examining the effects of factors on** ***Spartina*** **growth during the 2011 Mesocosm experiment.**
(DOCX)

Table S2 **ANOVA table examining the effects of factors on days to** ***Spartina*** **senescence during the 2012 Field experiment.**
(DOCX)
