# Supplementary figures and images for: Herbivore Impacts on Marsh Production Depend upon a Compensatory Continuum Mediated by Salinity Stress
Source: PLoS One. 2014 Oct 13;9(10):e110419. doi: 10.1371/journal.pone.0110419 (PMC4195738; doi:10.1371/journal.pone.0110419)

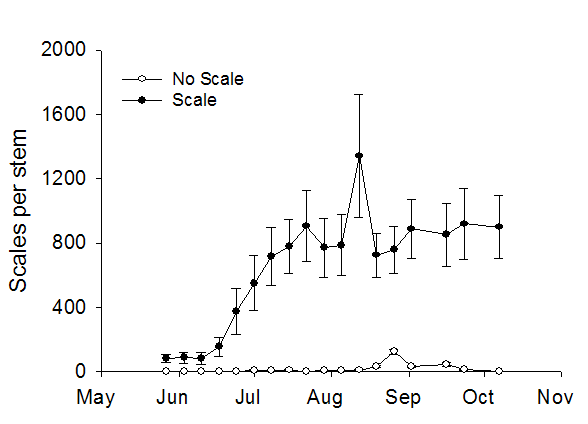

Supplement: Figure S1 — Densities of scales during the 2011 field experiment. Scales were removed from No Scale stems every 1–2 weeks. N = 10. Values are means ± SE. (TIF) [file pone.0110419.s001.tif]

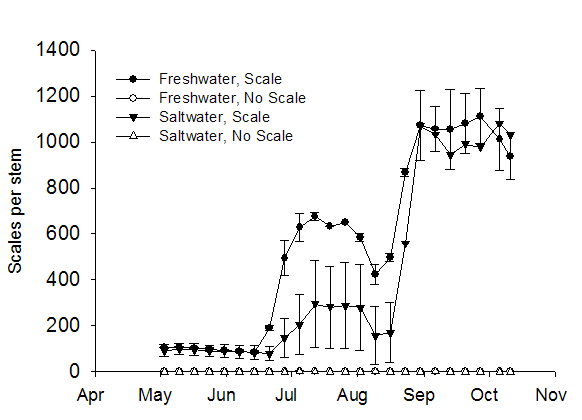

Supplement: Figure S2 — Densities of scales during the 2011 mesocosm experiment. Scales were removed from No Scale stems every week. N = 7–8. Values are means ± SE. (TIF) [file pone.0110419.s002.tif]

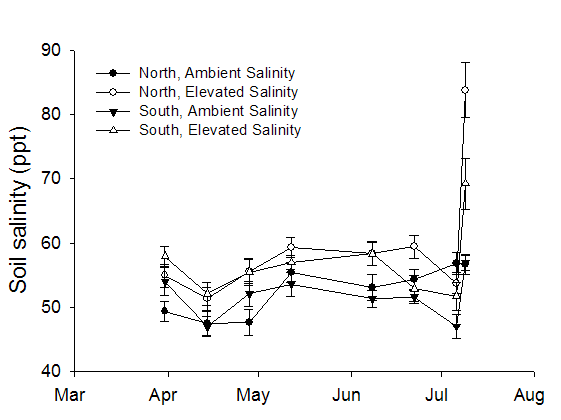

Supplement: Figure S3 — Soil salinities of plots during the 2012 field experiment. Because salinity did not depend upon Scale treatment, data were combined for Scale and no Scale stems to facilitate comparisons of the North and South sites. Soil salinity was always measured two weeks after salt additions, except on July 9 when sampling occurred three days after salt additions. Values are means ± SE. (TIF) [file pone.0110419.s003.tif]

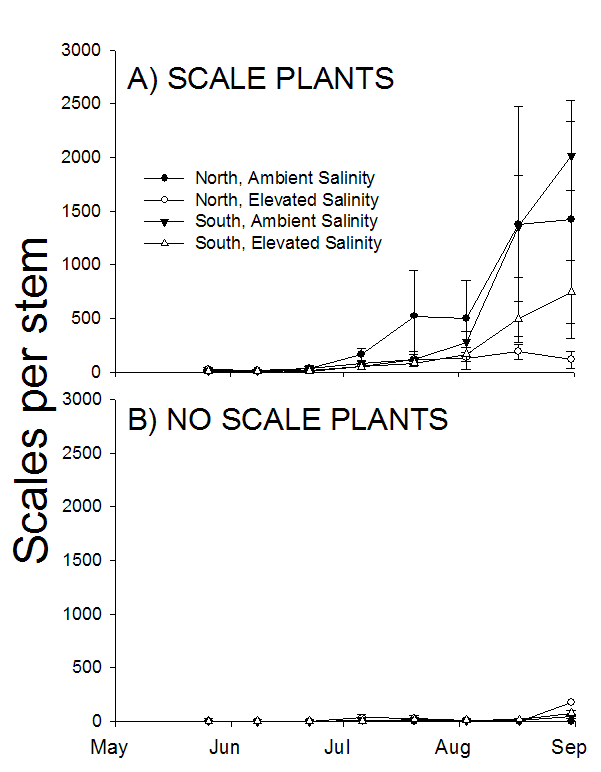

Supplement: Figure S4 — Densities of scales on stems during the 2012 field experiment. To highlight site and salt effects, data are shown separately for Scale (A) and No Scale (B) stems. Values are means ± SE. (TIF) [file pone.0110419.s004.tif]
