# Supplementary material for: Herbivore Impacts on Marsh Production Depend upon a Compensatory Continuum Mediated by Salinity Stress
Source: PLoS One. 2014 Oct 13;9(10):e110419. doi: 10.1371/journal.pone.0110419 (PMC4195738; doi:10.1371/journal.pone.0110419)
Supplement: Table S1 — ANOVA table examining the effects of factors on Spartina growth during the 2011 Mesocosm experiment. (DOCX) [file pone.0110419.s005.docx]

Table S1. ANOVA table examining the effects of factors on *Spartina* growth during the 2011 Mesocosm experiment.

Between Subjects

| Source | SS | df | MS | F | P |
| --- | --- | --- | --- | --- | --- |
| Salinity | 49.247 | 1 | 49.247 | 186.666 | <0.001 |
| Scale | 2.621 | 1 | 2.621 | 9.936 | 0.034 |
| Salinity x Scale | 2.675 | 1 | 2.675 | 10.141 | 0.033 |
| Error | 1.055 | 4 | 0.264 |  |  |

Within Subjects

| Source | SS | df | MS | F | P |
| --- | --- | --- | --- | --- | --- |
| Time | 35.851 | 1.525 | 1.434 | 242.425 | <0.001 |
| Time x Salinity | 22.345 | 1.525 | 0.894 | 151.097 | <0.001 |
| Time x Scale | 0.607 | 1.525 | 0.024 | 4.104 | 0.080 |
| Time x Salinity x Scale | 0.627 | 1.525 | 0.025 | 4.24 | 0.076 |
| Error | 0.592 | 6.1 | 0.006 |  |  |
