# Supplementary material for: Herbivore Impacts on Marsh Production Depend upon a Compensatory Continuum Mediated by Salinity Stress
Source: PLoS One. 2014 Oct 13;9(10):e110419. doi: 10.1371/journal.pone.0110419 (PMC4195738; doi:10.1371/journal.pone.0110419)
Supplement: Table S2 — ANOVA table examining the effects of factors on days to Spartina senescence during the 2012 Field experiment. (DOCX) [file pone.0110419.s006.docx]

Table S2. ANOVA table examining the effects of factors on days to *Spartina* senescence during the 2012 Field experiment.

| Source | SS | Df | MS | F | P |
| --- | --- | --- | --- | --- | --- |
| Salt | 469 | 1 | 469 | 0.523 | 0.473 |
| Scale | 476 | 1 | 476 | 0.531 | 0.470 |
| Site | 7043 | 1 | 7043 | 7.854 | 0.007 |
| Salinity x Scale | 2045 | 1 | 2045 | 2.281 | 0.137 |
| Salinity x Site | 469 | 1 | 469 | 0.523 | 0.473 |
| Scale x Site | 135 | 1 | 135 | 0.151 | 0.699 |
| Salinity x Scale x Site | 5181 | 1 | 5181 | 5.777 | 0.020 |
| Error | 45740 | 51 | 897 |  |  |
